# Alterations in the Gut-Microbial-Inflammasome-Brain Axis in a Mouse Model of Alzheimer’s Disease

**DOI:** 10.3390/cells10040779

**Published:** 2021-04-01

**Authors:** Pradeep K. Shukla, David F. Delotterie, Jianfeng Xiao, Joseph F. Pierre, RadhaKrishna Rao, Michael P. McDonald, Mohammad Moshahid Khan

**Affiliations:** 1 Department of Physiology, College of Medicine, University of Tennessee Health Science Center, Memphis, TN 38163, USA; rrao2@uthsc.edu; 2 Department of Neurology, College of Medicine, University of Tennessee Health Science Center, Memphis, TN 38163, USA; ddelotte@uthsc.edu (D.F.D.); jxiao@uthsc.edu (J.X.); mikemc@tennessee.edu (M.P.M.); 3 Department of Pediatrics, College of Medicine, University of Tennessee Health Science Center, Memphis, TN 38163, USA; jpierre1@uthsc.edu; 4 Department of Anatomy & Neurobiology, College of Medicine, University of Tennessee Health Science Center, Memphis, TN 38163, USA; 5 Neuroscience Institute, University of Tennessee Health Science Center, Memphis, TN 38163, USA; 6 Center for Muscle, Metabolism, and Neuropathology, Division of Rehabilitation Sciences and Department of Physical Therapy, College of Health Professions, University of Tennessee Health Science Center, Memphis, TN 38163, USA

**Keywords:** alzheimer’s disease, gut microbiota, NLRP3 inflammasome, amyloid-beta, neuroinflammation

## Abstract

Alzheimer’s disease (AD), a progressive neurodegenerative disorder characterized by memory loss and cognitive decline, is a major cause of death and disability among the older population. Despite decades of scientific research, the underlying etiological triggers are unknown. Recent studies suggested that gut microbiota can influence AD progression; however, potential mechanisms linking the gut microbiota with AD pathogenesis remain obscure. In the present study, we provided a potential mechanistic link between dysbiotic gut microbiota and neuroinflammation associated with AD progression. Using a mouse model of AD, we discovered that unfavorable gut microbiota are correlated with abnormally elevated expression of gut NLRP3 and lead to peripheral inflammasome activation, which in turn exacerbates AD-associated neuroinflammation. To this end, we observe significantly altered gut microbiota compositions in young and old 5xFAD mice compared to age-matched non-transgenic mice. Moreover, 5xFAD mice demonstrated compromised gut barrier function as evident from the loss of tight junction and adherens junction proteins compared to non-transgenic mice. Concurrently, we observed increased expression of NLRP3 inflammasome and IL-1β production in the 5xFAD gut. Consistent with our hypothesis, increased gut–microbial–inflammasome activation is positively correlated with enhanced astrogliosis and microglial activation, along with higher expression of NLRP3 inflammasome and IL-1β production in the brains of 5xFAD mice. These data indicate that the elevated expression of gut–microbial–inflammasome components may be an important trigger for subsequent downstream activation of inflammatory and potentially cytotoxic mediators, and gastrointestinal NLRP3 may promote NLRP3 inflammasome-mediated neuroinflammation. Thus, modulation of the gut microbiota may be a potential strategy for the treatment of AD-related neurological disorders in genetically susceptible hosts.

## 1. Introduction

Alzheimer’s disease (AD) is a progressive neurodegenerative disorder that accounts for the majority of dementia cases. An estimated 50 million people are living with dementia globally, and it is projected to approximately triple by 2050 [1]. As the population ages, AD will be expected to impose substantial health, social, and economic burdens on societies. Currently, there is no effective treatment that can prevent or slow the progression of AD. Several theories have been proposed to address the pathogenesis of AD, including neuronal loss, amyloid aggregation, synaptic dysfunction, tau hyperphosphorylation, oxidative stress, and inflammation [2,3,4]. Despite comprehensive research efforts, the molecular mechanisms underlying the onset and progression of AD in the aged population remain unclear. Emerging evidence from preclinical and clinical research suggests that gut microbiota may influence the pathogenic processes of several neurological diseases [5,6,7,8,9]. The alteration in gut microbiota composition could jeopardize host immune responses and promote the development of several neurological disorders including AD [10,11]. Thus, there is an unmet need to understand how alterations in the gut microbiota can be associated with AD pathogenesis.

The human gastrointestinal (GI) tract is populated by trillions of microbes that influence human physiology, synaptic function, immune function, gut barrier function, and host behavior, including cognition [10,12,13,14]. The gut microbiota can bidirectionally interplay with the central nervous system (CNS) through neuro–immuno–endocrinal signals which is termed the “gut–brain axis” [15]. Several neurotransmitters and metabolites, neurotrophic factors, and short-chain fatty acids (SCFAs) regulate several signaling pathways that in turn influence behavior, learning and memory, neuroinflammation, and neurodegeneration [16,17,18]. Although alterations in the gut microbiota composition have been documented in several gastrointestinal and metabolic diseases [19,20,21], the role of gut microbiota in neurodegenerative diseases, particularly AD, have only recently been recognized, even though the gut is often referred as the “second brain”. The composition and biodiversity of the gut microbiota of AD patients differ from those of healthy individuals [6,7]. Consistently, the gut microbiota composition of mouse models of AD is different from their control mice [9,22,23]. The gut microbiota composition changes with age in humans, with elderly people presenting a different profile from that of healthy adults [24]. Furthermore, several past studies have demonstrated that altered microbial composition could influence AD-related neuropathology and cognitive function [7,9,25]. Interestingly, manipulation of the gut microbiota effectively altered cognitive decline both in rodents and humans [26,27]. These findings strongly support the notion that alterations in gut microbiota composition may influence pathways toward neural damage and memory deficits in AD, whereas the introduction of “eubiotic” (normal) microbes or probiotics may prevent AD progression. Although these studies support the role of gut microbiota in influencing the disease condition, a targeted mechanistic link between gut microbiota and AD-associated neuroinflammation is lacking.

There is growing evidence supporting the role of a microbiota-gut-inflammasome-brain axis in neurodegenerative diseases [28,29,30]. Several studies have demonstrated that microbiota influence peripheral inflammatory response, which, in turn, could contribute to neuroinflammation and neurodegeneration [9,30,31,32]. NOD-like receptor protein 3 (NLRP3) inflammasome plays a significant role in both coordinating the host physiology and regulating the peripheral and central inflammatory responses in neurological diseases [31,33,34]. Upon sensing microbial or danger-associated molecular stimuli, the pyrin domain of NLRP3 interacts with the pyrin domain of the apoptosis-associated speck-like protein containing a caspase activating recruitment domain (ASC) to initiate the formation of the inflammasome complex, which consists of NLRP3, ASC, and procaspase-1 [30,35]. The activation of the inflammasome complex promotes the secretion of the proinflammatory cytokines interleukin (IL)-1β and IL-18 and induces pyroptosis [36]. However, there is currently insufficient evidence to link changes in the gut microbiota with the gut–microbial–inflammasome–brain axis in AD. In the present study, we sought to examine how changes in gut microbiota influence peripheral NLRP3 inflammasome, which in turn, might trigger neuroinflammation during disease progression using a transgenic mouse model of AD harboring five familial AD-related mutations (5xFAD). We provide compelling evidence demonstrating that gut dysbiosis occurs in young and old 5xFAD mice, associated with increased NLRP3 inflammasome in the brain. The significance of gut microbiota is emerging in the AD field and our study contributes to this growing body of evidence for microbial roles in AD pathogenesis that may provide new avenues to not only better understand AD pathophysiology but also lead to the development of novel microbiome-based therapies.

## 2. Materials and Methods

### 2.1. Mice and Brain Tissues

The mouse colony was established and sustained by periodically breeding male B6SJLF1/J wild-type animals with female hemizygous 5xFAD mice. All founders were initially purchased from the Jackson laboratory (stocks #100012 and #006554, respectively). The 5xFAD animals carry mutant human transgenes harboring two mutations in presenilin 1 (PSEN1; M146L and L286V) and three others in the amyloid precursor protein (APP; Swedish mutation KM670/671NL, Florida mutation I716V, London mutation V717I). As the two transgenes translocate together, the offspring either expresses both transgenes (5xFAD animals) or none of them (non-transgenic animals). Only male mice were used in the present experiments, preventing possible sex differences in gene and protein expressions. All mouse experiments were performed in accordance with the National Institutes of Health’s Guidelines for the Care and Use of Laboratory Animals and approved by the Institutional Animal Care and Use Committee (IACUC) at the University of Tennessee Health Science Center.

### 2.2. Microbiome Analysis

Colonic flushings of mice of each genotype and age were collected for microbiome analysis. DNA was extracted with TRIzol (Invitrogen, Carlsbad, CA, USA) as per the manufacturers’ instructions. 16S ribosomal RNA was analyzed by qPCR for different bacterial phyla or species using SYBR Green/ROX master mix (Qiagen) in an Applied Biosystems QuantStudio6 FlexReal-Time PCR instrument (Norwalk, CT, USA) as described in our published papers [37,38]. Primer sequences for 16S ribosomal RNA genes for Bacteroidetes, Firmicutes, *Bifidobacteria*, and *Lactobacillus* were detailed in Table S1.

### 2.3. Relative Quantitative Real-Time Reverse-Transcriptase PCR (RT-qPCR)

Total RNA was isolated from the colon by using Triazole kit (Invitrogen, Carlsbad, CA, USA) and quantified using NanoDrop described in our previous publications [39,40]. RNA (1.5 μg) was used for the generation of cDNAs using the ThermoScript RT-PCR system for first-strand synthesis (Invitrogen). Quantitative PCR (qPCR) reactions were performed using cDNA mix with primers and RT2 Real-Time SYBR Green/ROX master mix (Qiagen) in an Applied Biosystems QuantStudio 6 Flex Real-Time PCR instrument (Norwalk, CT, USA). The cycle parameters were 50 °C for 2 min, one denaturation step at 95 °C for 10 min, and 40 cycles of denaturation at 95 °C for 10 s followed by annealing and elongation at 60 °C. Mouse GAPDH was used as an endogenous control. Relative changes in gene expression were analyzed using 2^−ΔΔCT^ comparative method. The RT-qPCR primer sequences are listed in the Supplementary File (Table S1).

### 2.4. Immunofluorescence Staining

The immunofluorescent staining was performed as described previously by our group [40,41,42]. Briefly, mice were anesthetized with isoflurane, and the brain and colon tissues were removed quickly, postfixed in 4% paraformaldehyde solution, and cryoprotected with 30% sucrose in 0.1M phosphate-buffered saline (PBS) at 4 °C for 48 h. Brain tissue was sectioned on a cryostat at 25 μm. Sections were washed with PBS followed by blocking with 5% bovine serum albumin (BSA; Sigma Aldrich # A7906) and 0.3% Triton X-100. Brain sections were incubated overnight with monoclonal mouse NLRP3 (1:100; Adipogen #AG-20B-0014-C100), glial fibrillary acidic protein (GFAP; 1:500 polyclonal rabbit; Abcam #AB5804), and ionized calcium-binding adaptor molecule 1 (1:500; Iba-1; Wako). One of the following fluorescent secondary antibodies was used: Alexa Fluor 555 anti-rabbit, Alexa Fluor 488 anti-mouse, Alexa Fluor 488 anti-rabbit, or Alexa Fluor 555 anti-mouse (1:500, Life technologies, Grand Island, NY, USA). These sections were then washed and mounted using 4′,6-diamidino-2-phenylindole medium (DAPI, Vector laboratories). Images were captured with a Zeiss 710 laser scanning confocal microscope (Carl Zeiss GmbH, Jena, Germany) using a 40X oil objective with a total magnification of 400X. All images for tissue samples from different groups were collected and processed under identical conditions of laser, gain, and magnification.

Immunofluorescence staining in the colon was performed as described previously [38,40]. Colon sections were examined for components of the inflammasome (NLRP3, ASC, and caspase-1), F-actin, tight junction (TJ) proteins occludin and ZO1, and adherens junction (AJ) proteins E-cadherin, and β-catenin by confocal microscopy as described previously. Cryosections of the distal colon (10 μm thickness) were fixed in the acetone–methanol mixture (1:1) at 20 °C for 2 min and rehydrated in PBS. Sections were permeabilized with 0.3% Triton X-100 in PBS for 15 min and blocked with 5% non-fat milk in PBST for 1 h at room temperature. Sections were then incubated with primary antibodies for 2 h: rabbit polyclonal anti-ZO1, or mouse anti-occludin antibodies (BD Biosciences; San Jose, CA, USA), rabbit anti-β-catenin, or mouse monoclonal anti-E-cadherin antibodies (Thermo Fisher Scientific, Waltham, MA, USA); or NLRP3 (Adipogen); ASC (#AG-25B-0006-C100, Adipogen); or caspase-1 (# sc-514; Santacruz; Dallas, TX, USA), followed by incubation with appropriate secondary antibodies (1:100 dilution; Life technologies, Grand Island, NY, USA) for 1 h. Sections were coverslipped in mounting media with DAPI (ThermoFisher Scientific, Waltham, MA, USA). Images were captured with a Zeiss 710 laser scanning confocal microscope using a 20X with a total magnification of 200X. Immunofluorescence staining intensity was quantified by NIH Image J software (NIH, Bethesda, MD, USA) using three different fields per section of each mouse in the distal colon by an investigator blinded to the group assignment.

### 2.5. ELISA

Brain and colon tissues from the mice of each genotype and age were homogenized in a lysis buffer containing Halt™ protease and phosphatase inhibitor cocktail. A Sandwich ELISA Kit (R&D system; Minneapolis, MN, USA) was used to quantify IL-1β levels in the hippocampus and colon tissues of 5xFAD and non-transgenic mice according to the manufacturers’ instructions.

### 2.6. Western Blot Analyses

Brain and gut tissues were collected, flash-frozen, and stored at −80 °C. Methods for SDS-PAGE electrophoresis and Western blotting were adapted from our previous work [43]. The following primary antibodies were selected: mouse anti-NLRP3 (#AG-20B-0014-C100; Adipogen, San Diego, CA, USA), rabbit anti-Caspase-1 (#A0964; Abclonal, Woburn, MA, USA), or mouse anti-GAPDH (#AM4300; ThermoFisher Scientific, Waltham, MA, USA). Donkey anti-mouse or goat anti-rabbit HRP-labeled IgGs (Vector Laboratories, Burlingame, CA, USA) were used as secondary antibodies. Bands were detected by enhanced chemiluminescence using the SuperSignal™ West Femto substrate (Thermo-Fisher Scientific, Waltham, MA, USA). Membranes were imaged with the Odyssey Fc imaging system (Li-Cor, Lincoln, NE, USA). Signal density was quantified using the complementary software, Image Studio Lite (Li-Cor, Lincoln NE, USA), with the relative expression of NLRP3 and Caspase-1, normalized to endogenous GAPDH levels.

Two-way analysis of variance (ANOVA) was used to determine the effects of genotype and age on biochemical and morphological measures using GraphPad Prism 8 software (GraphPad Software, La Jolla, CA, USA). Values of *p* < 0.05 were considered statistically significant, and data are expressed as mean ± SEM.

### 2.7. Statistical Analysis

Two-way analysis of variance (ANOVA) was used to determine the effects of genotype and age on biochemical and morphological measures using GraphPad Prism 8 software (GraphPad Software, La Jolla, CA, USA). Values of *p* < 0.05 were considered statistically significant, and data are expressed as mean ± SEM.

## 3. Results

### 3.1. Altered Microbiome Composition in the Gut of Young and Old 5xFAD Mice

**Figure 1:**
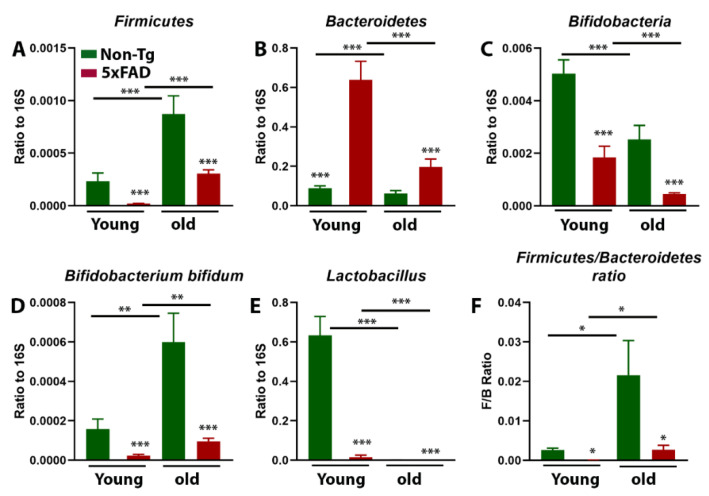
Age-dependent alteration in gut microbiota signature in 5xFAD mice. Firmicute (**A**), Bifidobacteria (**C**), *Bifidobacterium bifidum* (**D**), and *Lactobacillus* (**E**) were significantly depleted, while Bacteroidetes (**B**) were upregulated in colonic flushing in young (5-month) and old 5xFAD (15-month) mice compared to their age-matched control littermates. The F/B ratio is also altered in young and old 5xFAD mice compared to their age-matched non-transgenic (Non-Tg) mice (**F**). Values are expressed as means ± SEM. (N = 6–7/group) * *p* < 0.05, ** *p* < 0.01, *** *p* < 0.001.

To test the hypothesis that gut microbiota play an important role in AD pathology, we first compared the changes in gut microbiota signature in the fecal samples of age-matched 5xFAD mice and their control littermates. We found significantly altered expression of gut microbiota composition including Firmicutes, Bacteroidetes, *Bifidobacteria,* and *Lactobacillus* in colonic flushing of young (5 months) and aged (15 months) 5xFAD mice compared to non-transgenic (non-Tg) mice (Figure 1A–F). There was significant effect of age (F_1,22_ = 26.74, *p* = 0.0001) and genotype (F_1,22_ = 19.08, *p* = 0.0002) on Firmicutes, but no age*genotype interaction (F_1,22_ = 3.90, *p* = 0.06) (Figure 1A). There was a significant effect of age (F_1,22_ = 16.92, *p* = 0.0005) and genotype (F_1,22_ = 36.36, *p* < 0.0001) and an age*genotype interaction (F_1,22_ = 13.44, *p* = 0.0014) on the Bacteroidetes population (Figure 1B). For *Bifidobacteria* (Figure 1C), there was an overall effect of age (F_1,22_ = 22.13, *p* = 0.0001) and genotype (F_1,22_ = 40.22, *p* < 0.0001) but no significant age*genotype interaction (F_1,22_ = 1.832, *p* = 0.189). Similarly, there was a significant effect of age (F_1,22_ = 12.64, *p* = 0.0018) and genotype (F_1,22_ = 19.49, *p* = 0.0002) and an age*genotype interaction (F_1,22_ = 6.493, *p* = 0.018) on the *Bifidobacterium bifidum* strain level abundance (Figure 1D). There were significant effects of age (F_1,22_ = 52.75, *p* < 0.0001) and genotype (F_1,22_ = 48.60, *p* < 0.0001) and a significant age*genotype interaction (F_1,22_ = 48.06, *p* < 0.0001) on *Lactobacillus* (Figure 1E). Similarly, the Firmicutes to Bacteroidetes ratio (F/B ratio) was also significantly reduced in young and old 5xFAD mice (Figure 1F). We found significant effects of age (F_1,22_ = 6.98, *p* = 0.014) and genotype (F_1,22_ = 6.86, *p* = 0.015) but no significant effect of age*genotype interaction (F_1,22_ = 3.98, *p* = 0.058) on F/B ratio. Thus, our results support previous evidence that under conditions of neurodegeneration, the composition of gut microbiota is altered.

### 3.2. Compromised Gut Barrier Function in 5xFAD Mice

**Figure 2:**
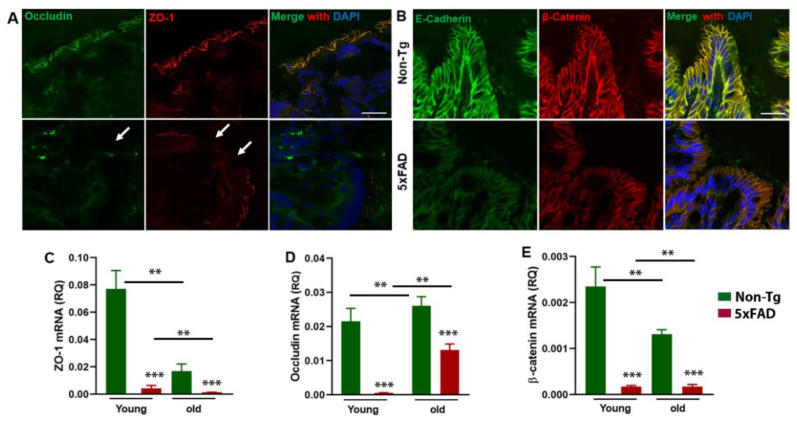
Compromised gut barrier function in 5xFAD mice. The upper panel shows representative images of distal colon sections showing the disruption of tight junction proteins (ZO-1 (red), occludin (green)) and adheren junction proteins (β-catenin (red), E-cadherin (green)) in young 5xFAD mice and their control littermates (**A**,**B**) N = 3/group. The lower panel (**C**–**E**) shows RT-qPCR analysis of junction proteins ZO-1, occludin, and β-catenin in young and old 5xFAD mice and their age-matched control littermates. The mRNA expressions of ZO-1, β- catenin, and occludin were significantly decreased in 5xFAD mice of both young and old ages. (N = 4/group). Values are expressed as means ± SEM. ** *p* < < 0.01, *** *p* < < 0.001.

The tight junction (TJ) proteins, including zonula occludens (ZO), occludin, and claudins, play a crucial role in maintaining the integrity of the intestinal tight junction barrier. An imbalance or loss of these proteins leads to compromised barrier integrity and is linked with several pathological conditions [44,45]. Therefore, we sought to determine whether changes in gut microbiota composition can impair the integrity of the gut barrier. For this purpose, the expression levels of mRNA and proteins involved in the maintenance of gut barrier integrity were quantified by RT-qPCR and immunohistochemistry (Figure 2A–E). To determine the effect of AD condition on colonic epithelial TJ and AJ integrity, distal colon cryosections from young 5xFAD and their age-matched non-Tg littermates were stained for TJ (occludin and ZO-1) and AJ (E-cadherin and β-catenin) proteins. The junctional distribution of occludin and ZO-1 was reduced (Figure 2A). Similarly, E-cadherin and β-catenin were also reduced in the distal colon of 5xFAD mice (Figure 2B). The mRNA expression levels for ZO-1, occludin, and β-catenin were markedly decreased in young and old 5xFAD mice compared to their non-Tg animals. There were significant effects of age (F_1,12_ = 13.91, *p* = 0.003) and genotype (F_1,12_ = 26.54, *p* = 0.0002) or the age*genotype interaction (F_1,12_ = 11.62, *p* = 0.005) on ZO-1 expression (Figure 2C). There were significant effects of age (F_1,12_ = 11.75, *p* = 0.005) and genotype (F_1,12_ = 46.66, *p* < 0.0001) on occludin expression (Figure 2D). Similar to ZO-1 data, there were significant effects of age (F_1,12_ = 9.86, *p* = 0.008), genotype (F_1,12_ = 73.42, *p* < 0.0001), and age*genotype interaction (F_1,12_ = 9.915, *p* = 0.0058) on β-catenin expression (Figure 2E). Taken together, these data indicate that the changes in gut microbial composition favor gut barrier disruption in 5xFAD mice which may lead to peripheral inflammation.

### 3.3. Increased NLRP3 Inflammasome and ASC Activation in the Gut of 5xFAD Mice

**Figure 3:**
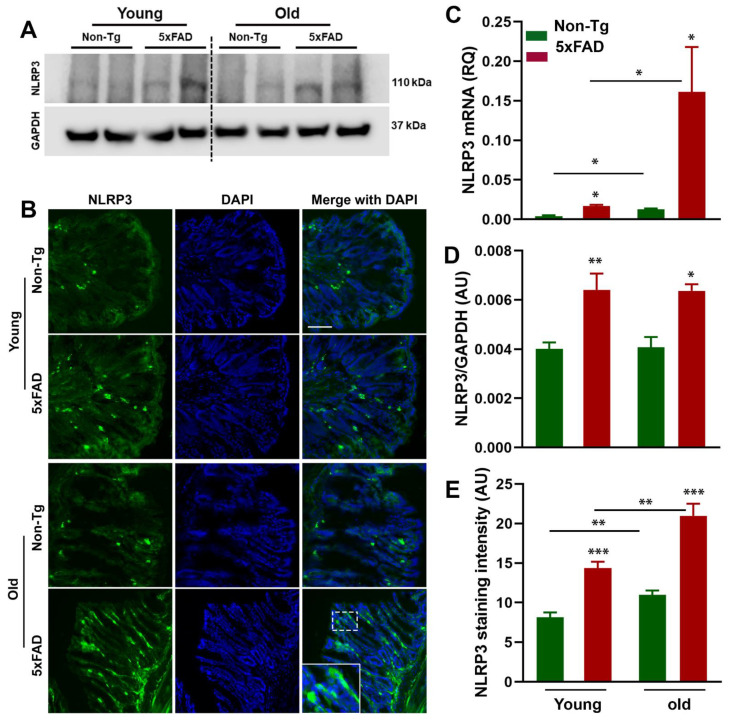
Age-dependent increased NLRP3 in the gut of 5xFAD mice. The expression levels of NLRP3 were determined by RT-qPCR (N = 4/group), Western blot (N = 3/group), and immunofluorescence (N = 3/group) in the colon of mice of each genotype and age (**A**–**E**). Western blot analysis of NLRP3 levels in the colon tissue of young and old Non-Tg and 5xFAD mice (**A**,**D**). The lower left panel shows representative fluorescent images of NLRP3 in colon (**B**). The right panel shows mRNA expression of NLRP3 (**C**) and quantitative analysis of NLRP3 (**E**) immunofluorescence. Values are expressed as means ± SEM. * *p* <0.05, ** *p* < 0.01, *** *p* < 0.001. Scale bar = 100 μm.

**Figure 4:**
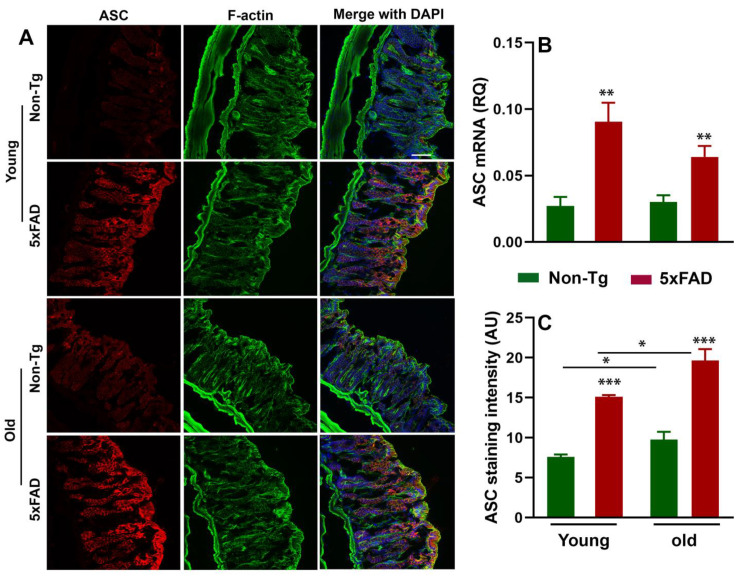
Age-dependent increased apoptosis-associated speck-like protein containing a caspase activating recruitment domain (ASC) in the gut of 5xFAD mice. The left panel shows representative fluorescent images of ASC colocalized with F-actin in the colon sections of young and old Non-Tg and 5xFAD mice (**A**). The right panel shows mRNA expression of ASC and quantitative analysis of ASC fluorescent intensity, respectively (**B**,**C**). Values are expressed as means ± SEM. N = 3 = 4/group. * *p* < 0.05, ** *p* < 0.01, *** *p* < 0.001. Scale bar = 100 μm.

It has been documented that microbiota interact with NLRP3 inflammasome in the gut, and the NLRP3 inflammasome has been shown to be activated in several neurodegenerative diseases [46,47,48]. Moreover, previous studies have reported that peripheral inflammation may influence CNS neuroinflammation during neuropathological conditions [27,49,50]. To determine whether the altered gut microbiota composition was accompanied by the activation of peripheral NLRP3 inflammasome and ASC, we first analyzed the NLRP3 inflammasome activation in the colon of young and old 5xFAD mice by RT-qPCR, Western blot, and immunohistochemistry. We observed increased mRNA and protein expression of NLRP3 in the colon tissue of young and old 5xFAD mice (Figure 3A–E). Two-way ANOVA analysis revealed that there were significant effects of age (F_1,12_ = 7.53, P = 0.017) and genotype (F_1,12_ = 7.80, *p* =0.016) on NLRP3 mRNA expression (Figure 3C). Consistent with the mRNA data, our Western blot (Figure 3A,D) and immunohistochemistry data (Figure 3B,E) showed significantly increased expression of NLRP3 in the colon sections of young and old 5xFAD mice compared to their age-matched control littermates. There were significant effects of age (F_1,8_ = 24.31, *p* = 0.001) and genotype (F_1,8_ = 71.59, *p* < 0.0001) on NLRP3 immunoreactivity. There was a larger effect of genotype on NLRP3 immunoreactivity than age. Similar to NLRP3 data, a higher expression of ASC (Figure 4A–C) was observed in the colon tissue of young and old 5xFAD as assayed by RT-qPCR and immunohistochemistry. Based on the two-way ANOVA analysis, there was a significant effect of genotype (F_1,12_ = 27.67, *p* = 0.002) but not age (F_1,12_ = 1.614, *p* = 0.228) on ASC mRNA expression. On the other hand, there were significant effects of age (F_1,8_ = 9.22, *p* = 0.016) and genotype (F_1,8_ = 50.86, *p* < 0.0001) on ASC immunoreactivity. These data indicate that the elevated expression of NLRP3 in the colon of 5xFAD mice is an important trigger for ASC recruitment and subsequent “downstream” activation of proinflammatory events, and gut microbiota could be a possible regulator of NLRP3 activation and inflammasome-mediated peripheral inflammation.

### 3.4. Caspase-1-Induced Inflammation in the Gut of 5xFAD Mice

**Figure 5:**
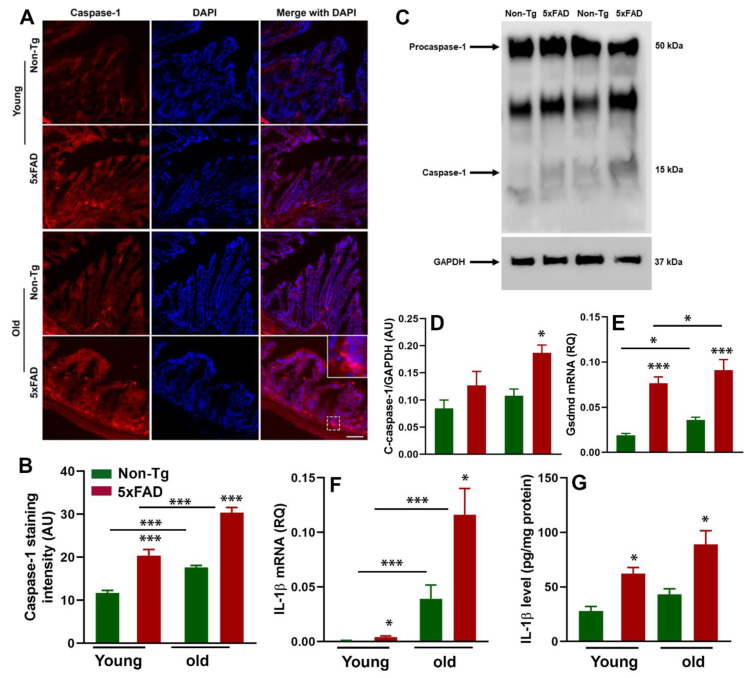
Age-dependent increased caspase-1 maturation, gasdermin-D, and IL-1β production in the gut of 5xFAD mice. Caspase-1 was quantified by immunofluorescence and Western blot (N = 3/group). The left panel shows representative fluorescent confocal images of caspase-1 and the quantification of their intensity in the colon sections of young and old 5xFAD mice and their age-matched control littermates (**A**,**B**) (N = 3/group; scale bar = 100 μm). The upper right panel shows Western blot analysis of cleaved caspase-1 levels in the colon of young and old 5xFAD mice and their age-matched control littermates (**C**,**D**). The middle right panel shows gasdermin-D mRNA expression levels in the colon of young and aged 5xFAD mice bt RT-qPCR (**E**). The right lower panels show quantification of interleukin (IL)-1β, by RT-qPCR (N = 4/group) and enzyme-linked immunosorbent assay (N = 6/group) (**F**,**G**), respectively, in the colon homogenates from mice of each genotype and age. Values are expressed as means ± SEM. * *p* < 0.05, *** *p* < 0.001.

NLRP3 activation triggers the formation of inflammasome via the self-oligomerization and the recruitment of adopter protein ASC and caspase-1, facilitating robust immune responses including the secretion of proinflammatory cytokines and pyroptosis [36,51]. Therefore, we investigated whether increased NLRP3 and ASC expression are positively correlated with caspase-1 mediated inflammatory response in the gut of the young and old 5xFAD mice. Our immunohistochemistry and Western blot data showed increased caspase-1 activation in the gut of 5xFAD mice compared to their age-matched non-transgenic littermates (Figure 5A–D). There was a robust effect of age (F_1,8_ = 64.14, *p* < 0.0001) and genotype (F_1,8_ = 115.4, *p* < 0.0001) on caspase-1 immunoreactivity. Given that immunohistochemistry did not differentiate procaspase 1 and cleaved caspase-1 expression, we quantified cleaved caspase-1 by Western blot. We observed increased cleaved caspase-1 which was more pronounced in aged 5xFAD mice (Figure 5C,D). It has been well documented that inflammasome formation caused the production of proinflammatory cytokine and pyroptosis [51]. Here, we sought to determine whether increased NLRP3 activation and subsequent inflammasome formation cause pyroptosis and IL-1β production in the colon tissue of 5xFAD mice. For this purpose, we examined gasdermine-D, a marker of pyroptosis and IL-1β production in the colon tissues of 5xFAD mice and their age-matched control littermates, by RT-qPCR and ELISA methods. There was a significant effect of age (F_1,16_ = 5.05, *p* = 0.039) and genotype (F_1,16_ = 63.34, *p* < 0.0001) on the mRNA expression level of gasdermin-D (Figure 5E). Next, we observed a significant difference in both RNA and protein levels of IL-1β in young and old 5xFAD mice compared to their age-matched control littermates (Figure 5F,G). There was a significant effect of age (F_1,12_ = 30.22, *p* = 0.0001) and genotype (F_1,12_ = 8.62, *p* = 0.012) on the mRNA expression level of IL-1β. In terms of IL-1β protein level, there was a significant effect of genotype (F_1,22_ = 7.12, *p* = 0.014) but not of age (F_1,22_ = 3.02, *p* = 0.096) or an age*genotype interaction (F_1,22_ = 0.12, *p* = 0.730). We speculate that NLRP3 activation further induces the upregulation of ASC with an increased expression of cleaved caspase-1, which further leads to the activation of the inflammatory response and pyroptosis.

### 3.5. Increased NLRP3 Inflammasome is Associated with Neuroinflammation in the Brains of 5xFAD Mice

**Figure 6:**
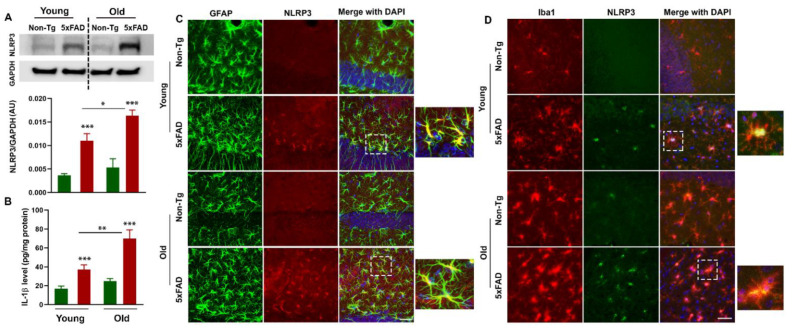
Age-dependent increased NLRP3 inflammasome-mediated neuroinflammation in the brains of 5xFAD mice. The left panel shows Western blot analysis of NLRP3 levels in the hippocampus of young and old 5xFAD mice and their age-matched control littermates (N = 3/group) (**A**). The left lower panel shows quantification of interleukin (IL)-1β, by ELISA (**B**) in the hippocampus of mice of each genotype and age. The values are expressed as mean ± SEM. * *p* < 0.05 (N = 6/group). The middle and right panels show representative confocal images of brain sections showing the colocalization of NLRP3 with astrocytic (glial fibrillary acidic protein (GFAP), green), and microglia (Iba1, red) markers (**C**,**D**). Concomitant expression of astrocytes and microglia with NLRP3 was more frequently observed in the hippocampus of young and old 5xFAD mice compared to control littermates. Scale bar = 50 μm. (N = 3/group). * *p* < 0.05, ** *p* < 0.01, *** *p* < 0.001.

Increased expression of NLRP3 has been documented in the blood and brain samples of cognitively impaired patients, suggesting peripheral NLRP3 inflammasome has a pathogenic role in AD [52,53]. We analyzed NLRP3, a component of the inflammasome, by Western blot in the hippocampus of young and aged 5xFAD mice and their non-transgenic littermate. We observed age-dependent increased NLRP3 levels in the hippocampus of 5xFAD mice as compared to non-transgenics. (Figure 6A). There were significant effects on age (F_1,8_ = 6.68, *p* = 0.032) and genotype (F_1,8_ = 45.83, *p* = 0.0001), but no effect of age*genotype interaction (F_1,8_ = 1.833, *p* = 0.213), on NLRP3 levels in the hippocampus of 5xFAD mice and their non-transgenic littermates. There is abundant evidence that microglia and astrocytes are activated in the brains of AD patients and secrete various pro-inflammatory cytokines that further exacerbate neurodegeneration [54,55]. Here, we examined the expression of astrocytes and microglia positive for NLRP3 in young and old 5xFAD mice and their control littermates (Figure 6C,D). 5xFAD mice contained more activated GFAP (green fluorescence) along with NLRP3 (red fluorescence)-positive astrocytes (Figure 6C). Additionally, increased activated Iba1 (red fluorescence) and NLRP3 (green fluorescence)-positive microglia (Figure 6D) with enhanced expression of NLRP3 was observed in 5xFAD mice as compared to non-transgenics. The NLRP3 inflammasome located in microglia and astrocytes triggers the production of proinflammatory cytokines such as IL-1β and induces pyroptotic cell death. Interestingly, increased NLRP3 activation in glial cells is positively correlated with the overproduction of IL-1β in the hippocampus of young and old 5xFAD mice compared to age-matched control littermates (Figure 6B). These data indicate that the elevated expression of NLRP3 in astrocytes and microglia in 5xFAD mice is an important trigger for subsequent downstream activation of the inflammatory response which may ultimately lead to AD-related neuropathology and memory loss.

## 4. Discussion

Prior research has demonstrated neuroinflammation plays a fundamental role in the progression of Alzheimer’s disease (AD). However, the role of peripheral inflammation, particularly originating from the gut, in AD progression is less well described. Growing evidence suggests that the dynamic changes in the gut microbiota may contribute to a broad range of neurological disorders through the release of metabolites, modulation of neuroimmune compartments, and activation of inflammatory pathways [7,14,56,57]. Among these pathways, studies showed that the gut–microbial–NLRP3 inflammasome activation plays a critical role in neuroinflammation and AD-related neuropathology. However, the nature of the mechanisms through which alterations in gut microbiota composition can influence neuroinflammation in AD is not known. In this study, we provided cogent mechanistic evidence supporting the role of gut dysbiosis in impairment of gut barrier function and activation of peripheral inflammation, which in turn may trigger neuroinflammation in a mouse model of AD. We specifically find that elevated Bacteroidetes with concurrent loss of protective bacteria, such as *Bifidobacterium*, are positively correlated with compromised gut barrier function and increased gut inflammasome activation in 5xFAD mice. We further report that increased peripheral inflammasome activation triggers NLRP3-mediated neuroinflammation in the brains of 5xFAD mice. The observation that the gut microbiota and peripheral inflammation promote neuroinflammation during neurodegenerative conditions is consistent with previous reports [30,57,58].

In our study, we chose the 5xFAD transgenic mice because they demonstrate robust neuropathological alterations including Aβ deposition (Figure S1) comparable to those observed in AD patients. To determine whether gut dysbiosis is associated with AD, we examined the gut microbiota in fecal samples of young and old 5xFAD mice and their non-transgenic littermates. We found altered gut microbiota composition in the fecal samples of young and old 5xFAD mice as compared to their age-matched non-transgenic littermates. In particular, the relative abundance of *Firmicutes, Bifidobacteria,* and *Lactobacillus* is decreased while the relative abundance of Bacteroidetes is significantly increased in the fecal samples of 5xFAD mice. However, microbiota from 5xFAD and their control littermates showed notable differences across ages in the same genotype. Our findings are consistent with previous studies that showed significant alterations in the composition and biodiversity of the gut microbiota in preclinical AD models and AD patients [6,7,9,59]. For instance, Vogt et al. found a significant alteration in gut microbiota composition, including decreased Firmicutes, increased Bacteroidetes, and decreased Bifidobacterium in the fecal samples of AD patients, compared to the microbiota of healthy controls, suggesting AD is associated with changes in the gut microbiome [7]. Similar to this study, Zhuang and colleagues also found altered gut microbial composition in the fecal samples of AD patients compared to age- and sex-matched cognitively normal controls [6], suggesting the potential contribution of gut microbiota to AD pathogenesis. Consistent with human studies, Bäuerl et al. reported a significant shift in the gut microbiota composition during AD progression in an APP/PSS1 transgenic mouse model of AD [23]. Chen and coworkers found a significant alteration of the gut microbiota composition in two different mouse models of AD (5xFAD and 3xTg mice), indicating the potential influence of gut microbiota in AD pathogenesis [60]. Contrary to a previous study [8], in our hands, the firmicutes and bacteroidetes ratio was significantly reduced in the fecal samples of 5xFAD mice compared to non-transgenic mice, presumably due to different age groups, diet, and environmental factors. Similarly, a study by Kim and coworkers demonstrated the altered composition of gut microbiota that are correlated with the loss of epithelial barrier integrity and chronic intestinal and systemic inflammation in mice with AD-related neuropathology [9]. In support of this idea, a study by Harach and coworkers showed that germ-free APPPS1-21 transgenic mice, expressing familial AD-linked APPSWE and PS1L166P transgenes, display more reduced Aβ deposition than conventional transgenic mice [61], indicating that gut microbiota influence the development of amyloid pathology. Consistent with this notion, a study by Lee et al. demonstrated that probiotic treatment modulates gut dysbiosis and gut barrier function and suppresses the accumulation of Aβ plaques, gliosis, and cognitive deficits, suggesting that maintaining gut microbial homeostasis may have beneficial effects on AD [26]. Therefore, it is reasonable to infer that alterations in the gut microbial composition may lead to pathophysiological changes in the brain of 5xFAD mice.

Gut barrier function is critical for normal gut homeostasis, and a compromised gut barrier function is closely associated with the pathophysiology of gut inflammation [62,63]. Gut microbiota interact directly with the gut epithelial cells, which, together with the mucus layer, act as a barrier interposed between the luminal contents and the underlying neuroimmune compartments. Proteins associated with tight junction and adheren junction, such as zonula occludin (ZO), occludin, E-cadherin, and β-catenin, contribute to the formation of gut tight junction barrier that ensures the epithelial barrier integrity and regulate the paracellular permeability [64]. Gut microbiota and their metabolic products, such as short-chain fatty acids (SCFAs), regulate the integrity of the intestinal epithelial barrier [62]. Bacterial metabolites also act as epigenetic modifiers such as butyrate and influence barrier integrity and brain function. For instance, *eubacterium rectale* is known to produce butyric acid, a SCFA and has already been associated with cognitive decline [52,65]. Moreover, it has been reported that an altered gut microbiota composition can influence gut barrier dysfunction, which facilitates the translocation of gut bacteria-derived pathogens and toxins such as lipopolysaccharide (LPS) into the systemic circulation. Consistent with this notion, recent studies showed gut barrier dysfunction and increased gut permeability in mouse models of AD and dementia patients [66,67,68]. Moreover, the integrity of the epithelial gut barrier is reduced with aging and neurodegenerative conditions [69,70,71]. Consistent with these findings, our RT-qPCR and immunohistochemistry data showed a significantly reduced expression of tight and adheren junction proteins in the colon tissues of 5xFAD mice compared to their age-matched control littermates, suggesting compromised gut barrier function in AD condition. Our findings of compromised gut barrier function in a mouse model of AD are in good agreement with those studies which reported gut barrier dysfunction associated with altered microbial composition during neurodegenerative conditions [60,72]. Given that gut microbiota can influence the rearrangement of TJs which mediate gut permeability [73], dysbiosis observed in this study could drive increased gut barrier dysfunction observed in 5xFAD mice. Commensal bacteria and probiotics have been shown to enhance intestinal barrier integrity. For example, *Lactobacillus plantarum* and *Bifidobacterium infantis* enhance the intestinal barrier by increasing the expression of the protein associated with tight junctions [73]. On the contrary, the exotoxin of *Bacteroides fragilis* disrupts adherens junctions by cleavage of cell adhesion molecules such as E-cadherin [74]. These findings highlight the significance of gut microbiota in the maintenance of gut barrier function. We speculate that an imbalance in gut microbiota signature and compromised intestinal barrier structure may flare up into an overwhelming immune response in the gut milieu.

Although the role of the gut microbiome in neurodegenerative diseases is fairly well studied, the mechanisms of its involvement in neuroinflammation are less understood. Gut microbiota can contribute to inflammation in several ways, including direct inflammatory stimulation, the production of pro-inflammatory metabolites, and the loss of immune regulatory function [10,75]. It has been demonstrated that gut microbiota release a significant level of amyloids and LPS and trigger enteric and peripheral inflammatory responses, which, in turn, could contribute to neuroinflammation [26,76]. For instance, the plasma concentrations of LPS in AD patients and mouse models of AD are significantly higher than in their respective controls [26,77]. Interestingly, probiotic treatment suppresses LPS production and ameliorates inflammation and Aβ deposition [26]. Besides, microbial products can directly activate circulating immune cells, which, in turn, infiltrate the CNS and alter brain physiology [14,49,78]. The aggregation of a large number of inflammatory mediators can further exacerbate glia activation and induce neural degeneration, ultimately impairing memory and cognitive functions. A study by Wang et al. demonstrated that changes in gut microbiota signature facilitate the infiltration of peripheral immune cells in the brain, resulting in enhanced microglial activation that contributes to Aβ burden and cognitive impairment in mice [11]. Most of the innate immune responses are mediated by pattern recognition receptors such as Toll-like receptors (TLRs) or cytosolic NOD-like receptors (NLRs) that recognize the microbe-associated molecular patterns expressed by gut microbiota. NOD-like receptor protein 3 (NLRP3) inflammasome serves as an important player in both coordinating the host physiology and regulating the peripheral and central inflammatory responses in neurological diseases [31,33,47]. Upon activation, NLRP3 triggers the assembly of inflammasome via the self-oligomerization and the recruitment of adopter protein ASC and pro-caspase-1, facilitating robust immune responses including the secretion of proinflammatory cytokines and pyroptosis [48,79]. The activated NLRP3 inflammasome is a critical driver of neuroinflammation in AD as shown by several past studies [48,80]. In this context, there is growing amount of evidence supporting the occurrence of the dynamic interplay between the gut microbiota and the NLRP3 inflammasome, currently referred to as a microbiota-gut-inflammasome-brain axis, where gut microbiota modulate peripheral inflammatory pathways through inflammasome signaling that, in turn, contribute to and/or influence CNS neuroinflammation [29,30,34,52,58]. NLRP3 recognizes the microbe-associated molecular patterns expressed by gut microbiota, and changes in microbial composition are associated with increased levels of NLRP3 in the plasma of patients with cognitive impairment and brain amyloidosis [52]. Moreover, the microbial product LPS has also been reported to trigger NLRP3 inflammasome [81]. Direct evidence of microbiota-gut-inflammasome-brain axis comes from a study by Shen et al., which shows that fecal transplantation from AD patients in APP/PS1 transgenic mice induces the activation of gut NLRP3 inflammasome and the production of proinflammatory cytokines, which further aggravate neuroinflammation and cognitive deficits [30]. Interestingly, they further observed that the introduction of youthful microbiota alters the inflammatory response in the mouse intestinal and brain tissues. A study by Lowe and coworkers showed that the depletion of gut microbiota with an antibiotic cocktail influenced the inflammasome signaling in the intestine, circulation, and the brain [34], suggesting that the gut microbiota can influence the expression of inflammasome components elsewhere. Indeed, microbiota in NLRP3-deficient mice differ from those in wild-type animals [82]. Interestingly, fecal microbiota transplantation (FMT) from NLRP3-deficient mice significantly ameliorated depressive-like behavior and locomotor activities [83]. Therefore, it is conceivable that the changes in gut microbiota can influence neuroinflammation by activating the gut NLRP3 inflammasome and releasing inflammatory mediators. Compared to the findings from previous reports, our study further clarifies the correlation between gut microbiota and neuroinflammation in a transgenic model of AD. In the present study, we also found that 5xFAD mice exhibited age-dependent increased NLRP3 inflammation activation in the gut tissues along with increased expression levels of ASC,caspase-1, and gasdermin-D, which are positively correlated with increased production of proinflammatory cytokine IL-1β. Interestingly, increased peripheral inflammasome activation is positively correlated with activated microglia and astrogliosis in the hippocampus of young and aged 5xFAD mice. Our findings are in agreement with previous studies that peripheral inflammation triggers a neuroinflammatory response, characterized by sustained glial activation with detrimental consequences for learning and memory in neurodegenerative condition [30,52,84,85,86,87], thus supporting the view that the gut–microbial inflammatory response might represent the earliest events of neuroinflammation in AD. In support of these studies, a recent study by Tjera et al. shows that systemic inflammation impairs microglia Aβ clearance through NLRP3 inflammasome in a mouse model of AD [81], suggesting a contributory role of peripheral inflammation in the induction of NLRP3-driven neuroinflammation.

There are several limitations of this study. First, we have not utilized the whole genome shotgun sequencing method to identify the gut microbiota signature at species and strain levels that may account for AD-associated neuroinflammation. Therefore, further study is required to identify the specific gut microbes during AD onset and progression which may yield novel therapeutic and diagnostic biomarkers. Second, although we did see a change in NLRP3 inflammasome in the gut and the brain tissues of 5xFAD mice, it remains unknown whether the peripheral inflammasome is sufficient to influence AD-related neuropathology and cognitive function. These limitations encourage further works to clarify the mechanism of the microbiota-gut-inflammasome-brain axis and their functional impact on AD pathogenesis. In conclusion, our findings lead to the speculation that an original shift in gut microbiota signature alters the integrity of gut barrier function through the release of metabolites and triggers the pathological activation of the gut and subsequently brain inflammasome complexes, ultimately leading to exacerbated neuroinflammation in a mouse model of AD.

## Data Availability

The data generated and analyzed in this study are available from the corresponding authors on reasonable request.

## Supplementary Materials

The following are available online at https://www.mdpi.com/article/10.3390/cells10040779/s1, Table S1: Mouse primers for qPCR and RT-qPCR and Figure S1: Aβ deposition in the hippocampus of 5xFAD mice and their control littermates.
