# Supplementary material for: Alterations in the Gut-Microbial-Inflammasome-Brain Axis in a Mouse Model of Alzheimer’s Disease
Source: Cells. 2021 Apr 1;10(4):779. doi: 10.3390/cells10040779 (PMC8067249; doi:10.3390/cells10040779)
Supplement: Supplementary file 1 [file cells-10-00779-s001.pdf]

Table S1: Mouse primers for qPCR and RT-qPCR

Occludin	Forward: TTGAAAGTCCACCTCCTTACAGA
Occludin	Reverse: CCGGATAAAAAGAGTACGCTGG
ZO-1	Forward: GCCGCTAAGAGCACAGCAA
ZO-1	Reverse: TCCCCACTCTGAAAATGAGGA
β-catenin	Forward: ATGGAGCCGGACAGAAAAGC
β-catenin	Reverse: CTTGCCACTCAGGGAAGGA
NLRP3	Forward: TCATGTTGCCTGTTCTTCCA
NLRP3	Reverse: CCGGTTGGTGCTTAGACTTG
ASC	Forward: CTTGTCAGGGGATGAACTCAAAA
ASC	Reverse: GCCATACGACTCCAGATAGTAGC
Gasdermin D	Forward: CCATCGGCCTTTGAGAAAGTG
Gasdermin D	Reverse: ACACATGAATAACGGGGTTTCC
IL-1β	Forward: GCAACTGTTCCTGAACTCAACT
IL-1β	Reverse: ATCTTTTGGGGTCCGTCAACT
16S rDNA, (universal) UniF334-F	Forward: ACTCCTACGGGAGGCAGCAGT
16S rDNA, (universal) UniR514	Reverse: ATTACCGCGGCTGCTGGC
16S rDNA (Firmicutes) 928F-Firm	Forward: TGAAACTYAAAGGAATTGACG
16S rDNA (Firmicutes) Firm1040R	Reverse: ACCATGCACCACCTGTC
16s rDNA (Bacteroidetes) 798cfbF	Forward: CRAACAGGATTAGATACCCT
16s rDNA (Bacteroidetes) cfb967R	Reverse: GGTAAGGTTCCTCGCGTAT
Lactobacillus	Forward: CCACCTTCCTCCGGTTTGTCA
Lactobacillus	Reverse: AGGGTGAAGTCGTAACAAGTAGCC
Bifidobacterium	Forward: CCCTGGAAAGGGTGG
Bifidobacterium	Reverse: GGTGTTCTTCCCGATATCTACA

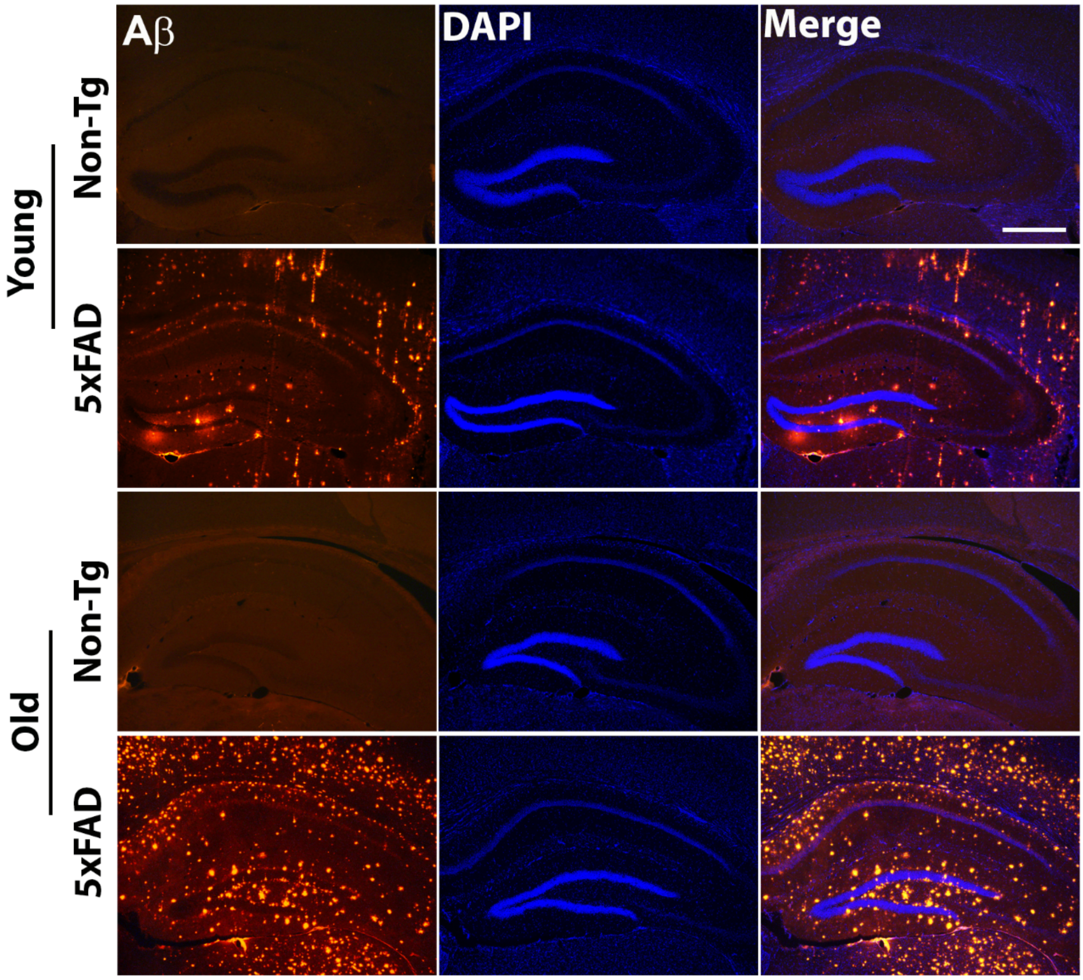

Figure S1. Representative images of Aβ deposition (BAM10; red) in the hippocampus of young and old 5xFAD and their control littermates. Scalebar- 500µM N=3 mice/group.
